# Development of Antimicrobial Defined Daily Dose (DDD) for the Pediatric Population

**DOI:** 10.3390/antibiotics12020276

**Published:** 2023-01-31

**Authors:** Elena Montecatine-Alonso, Marta Mejías-Trueba, Walter Alfredo Goycochea-Valdivia, Estibaliz Chavarri-Gil, Cecilia M. Fernández-Llamazares, Elisenda Dolz, José María Gutiérrez-Urbón, Carmen Gallego-Fernández, Jesús Llorente-Gutiérrez, María Victoria Gil-Navarro

**Affiliations:** 1 Department of Pharmacy, Hospital Universitario Virgen del Rocio, 41013 Seville, Spain; 2 Department of Infectious Diseases, Microbiology and Preventive Medicine, Infectious Diseases Research Group, Institute of Biomedicine of Seville (IBiS), University of Seville/Spanish National Research Council/University Hospital Virgen del Rocio, 41013 Seville, Spain; 3 Paediatric Infectious Diseases, Rheumatology and Immunology Unit, Hospital Universitario Virgen del Rocío, Institute of Biomedicine of Seville (IBIS), 41013 Sevilla, Spain; 4 Department of Pharmacy, Hospital Universitario de Cruces, 48903 Barakaldo, Spain; 5 Department of Pharmacy, Hospital Gregorio Marañón, Instituto de Investigación Sanitaria Gregorio Marañón, Research Network on Maternal and Child Health and Development II (Red SAMID II). Spanish Health Institute Carlos III, 28007 Madrid, Spain; 6 Department of Pharmacy, Complejo Hospitalario Universitario Insular-Materno Infantil, 35016 Las Palmas de Gran Canarias, Spain; 7 Department of Pharmacy, Complexo Hospitalario Universitario A Coruña, 15006 A Coruña, Spain; 8 Department of Pharmacy, Hospital Regional Universitario de Málaga, 29010 Málaga, Spain; 9 Department of Pharmacy, Hospital Universitario Infanta Sofía, 28703 San Sebastián de los Reyes, Spain

**Keywords:** pediatric, antimicrobials, defined daily dose (DDD), antimicrobial stewardship program

## Abstract

Antimicrobial stewardship programs (ASPs) optimize antimicrobial use, improve patient outcomes, and reduce resistance. To assess the effectiveness of ASPs, it is necessary to have indicators that can be widely used. Defined daily dose (DDD) was designed by WHO for the adult population as a consumption indicator. However, there are no DDDs adapted to the pediatric population. The main objective of this study is to establish the most appropriate DDD values in this population. An observational, retrospective, multicenter study was conducted. Antimicrobial prescriptions were collected from pediatric wards of seven Spanish tertiary hospitals for 2 years. The DDDs obtained from the prescriptions were compared with the theoretical DDDs agreed upon in the first stage. To select the optimal DDD, the following were analyzed: power value, magnitude obtained from the differences in the DDD, statistical significance, and degree of agreement in the stipulated doses. A total of 4788 prescriptions were collected. Pediatric DDD was defined for 30 different antimicrobials. A potency >80% was obtained in 24 antibiotics. 51.2% of the selected DDD correspond to Phase I and 39.5% from Phase II. Pediatric DDD of different antimicrobials was obtained, providing an indicator that can be used globally in different hospitals to analyze the consumption and efficacy of ASPs.

## 1. Introduction

Antimicrobial agents are among the most commonly prescribed medications, especially in children and neonates [1]. It has been demonstrated that between 20 to 50% of these prescriptions are potentially unnecessary or inappropriate [2]. Judicious use of antibiotics is essential to slow the emergence of antibiotic resistance in bacteria and extend the useful lifetime of effective antibiotics [3].

Antimicrobial stewardship programs (ASP) are a multidisciplinary institutional initiative focusing primarily on the improvement of antimicrobial prescribing practices, limiting inappropriate use and curtailing the rise of antimicrobial resistance [4].

ASP monitor compliance with interventions that aim to optimize therapy and identify utilization patterns that warrant further investigation [4,5]. Their clinical and ecological benefits have been demonstrated in hospitals as well as in the community [6,7].

Defined daily dose (DDD) is one of the established metrics used by ASP, allowing the assessment of antimicrobial consumption. The World Health Organization (WHO) expresses DDD as the average standard daily dose of a drug used in a 70 kg adult for the most common indication [8,9]. However, the validity of the DDD WHO definition is questionable in children, in which dose recommendations vary according to age and body weight [8,10,11].

Although other metrics not influenced by weight or age variability can be used in children, DDD remains easier to measure and more accessible for many centers. In view of the need to have a metric better adjusted to this population’s prescription particularities, a useful method for antimicrobial DDD measurement in pediatrics was designed (KiDDDs project) [12]. For this purpose, a multicenter observational study was carried out to obtain the theoretical DDD. 

The main objective of this study is to validate the theoretical DDD obtained from the method developed for its use in pediatric ASP and to establish the most appropriate DDD values to be used in clinical practice in this population.

## 2. Materials and Methods

### 2.1. Study Design

This is an observational, retrospective, multicenter study consisting of two phases.

The first phase was aimed at the theoretical calculation of pediatric DDD. For this purpose, the age of children admitted into 10 Spanish hospitals was considered, and the doses for each antimicrobial in its most common indication were established using the Delphi method. Theoretical weight (kg) for DDD calculation was selected for the obtained median age by sex using the 50th percentile according to the WHO weight for age graphs in pediatrics. DDD (g) for each antimicrobial was calculated by multiplying the weight obtained by the antimicrobial dose agreed upon by the expert panel (mg/kg). Pediatric DDD was designed for 44 antimicrobials (29 administered intravenously and 15 orally). The results of this first phase have been published elsewhere [12].

The second phase, detailed in this manuscript, constitutes the validation of the study. Antimicrobial prescriptions were collected from pediatric wards of 7 Spanish tertiary hospitals over a 2-year period (2017 and 2018).

### 2.2. Data Collection

Prescriptions data were collected using weekly prevalence cut-offs. A researcher from each hospital collected data through the electronic prescribing systems of each center or by visiting the pediatric units in those hospitals with manual prescriptions. All variables studied were stored in an electronic data collection notebook: demographic variables (age, gender and weight) and antimicrobial used (active substance, dose, frequency and route of administration). Those antimicrobials included in the first stage of our project were considered.

From the data collected, the total dose of antibiotic received per patient (mg/day) was calculated, and subsequently, the median of the resulting DDD per antibiotic (g/day) was obtained. These DDD obtained from actual prescriptions (Phase II) were compared with the theoretical DDD (Phase I) agreed by the Delphi Method at first stage.

### 2.3. Data Analysis

A detailed examination of all cases was performed using a combination of proportions and percentages for qualitative variables and means and standard deviations or medians and quartiles for quantitative variables. These calculations were based on whether the variables followed a normal distribution. The study also included the calculation of population confidence intervals at 95% (CI 95%), given the large sample size, assuming compliance with normal distribution for quantitative variables, and the application of the central limit theorem for qualitative variables. 

To describe the DDD of each antibiotic obtained in Phase II, the median and its CI 95% were used, and the difference observed between phases was analyzed using the Wilcoxon nonparametric test. The power value and the *p*-value were calculated for each Wilcoxon test performed, with a Type I error of 1%. All statistical analyses were performed using R version 4.0.3 [13].

### 2.4. DDD Selection Criteria

The selection of the optimal DDD value for each antimicrobial considers the power value, the magnitude obtained from the differences in the DDD medians between both phases, the statistical significance obtained by the Wilcoxon test, and the degree of agreement in the doses used for the DDD calculation in Phase I. 

Antimicrobials with >80% power were evaluated. Of these, Phase I or II DDDs were selected based on the results of the Wilcoxon test and the magnitude of the differences in the DDDs between both bases fundamentally. A high degree of consensus on the established dose (≥75%) was also valued positively, although it was considered a conditional criterion. 

For the rest of the antimicrobials whose power was ≤80%, statistically, we cannot say if there are differences between Phase I and Phase II DDD, but in those where there was a consensus ≥75%, we could use Phase I DDD with caution. The selection criteria are shown in Table 1.

### 2.5. Ethics

The study was approved by the Spanish Agency for Medicines and Sanitary Products. It was classified as a “post-authorization study with other designs different from prospective design” on 11 May 2015 (ID number: GAT-TEI-2015-01). Subsequently, it was approved by the Ethics Committee of the Virgen del Rocío-Macarena University Hospital on 24 October 2016 (ID number: 0620-N-15). The study was conducted in accordance with the Declaration of Helsinki of the World Medical Association. 

## 3. Results

A total of 4788 prescriptions were collected. The median age and weight were 6.64 (SD: 4.6) years and 25.7 (SD: 16.78) kilograms, respectively. 58.9% of prescriptions were for boys. The 34 selected antimicrobials and the number of prescriptions for each of them are shown in Table 2.

The five most prescribed antimicrobials were amoxicillin-clavulanic (20.9%), gentamicin (9.5%), metronidazole (8.8%), cefazolin (8.4%) and cefotaxime (5.3%). The intravenous route of administration was the most commonly used (86.5%).

Table 3 (intravenous antimicrobials) and Table 4 (oral antimicrobials) show data from Phase I (the DDD value and the degree of consensus on the dose used for the calculation of the Phase I DDD), data from Phase II (the median of the DDD resulting in Phase II with its CI 95% for each antimicrobial) and the value of the difference of the DDD between both phases. The value of the DDD finally selected is also shown and is summarized in the Supplementary material (Table S1). 

A total of 16 out of 29 intravenous antimicrobials and 8 out of 14 oral antimicrobials had a power >80%. The results related to the selection of the DDDs for each antibiotic (both intravenous and oral) based on the established criteria are summarized in Table 5. 

Penicillin G and fosfomycin had to be excluded from the analysis because there was an error in data collection in Phase II, making it impossible to compare the DDD with that calculated in Phase I.

## 4. Discussion

This study defined the DDD of antimicrobials in the pediatric population, comparing the theoretical DDD obtained in Phase I of the KiDDDs project [12] with the DDD calculated from actual prescriptions in hospitalized pediatric patients. To our knowledge, this is the first study aimed at defining and validating DDD for the pediatric population.

Focusing on the evidence published so far, the best metric for the evaluation of the aggregate consumption of antimicrobials in pediatrics has not yet been defined. Although days of therapy (DOT) seems to be the most currently recommended metric in hospital settings despite its low feasibility [14], other accepted methods are available like prescribed daily doses (PDD), point prevalence surveys (PPS), length of therapy (LOT) or DDD [15,16,17]. D’Amore et al. used different metrics to assess the use of antibiotics in hospitalized pediatric patients [18], evaluating DOT, PDD and LOT and comparing PDD with DDD. In this study, the authors determined that PDD increased with age and approached DDD only in children aged ≥10 years, concluding that DOT, LOT, and PDD are better alternatives to DDD in children. However, this is logical since the DDD values used are those stipulated for the adult population, which differ greatly from those obtained for the pediatric population.

The European Project on Antibiotic Resistance and Prescription in Children (ARPEC) and the European Surveillance of Antimicrobial Consumption (ESAC) have used PPS to monitor antimicrobial consumption in children [16,17,18,19]. This metric can assess antimicrobial consumption over short periods of time using retrospective, prospective, or mixed designs [17,18,19]. However, data from specific PPS time points are susceptible to case mix complexity, seasonality, and sample variability, as we mentioned in the previous work. [12]. 

Some authors favor the use of DDD in pediatrics. Nitsch-Osuch et al. [20] assessed antibiotic consumption by calculating the DDDs per 100 patient days and DDDs per 100 admissions in the Special Neonatal Care Unit. Liem et al. [21], Porta et al. [22] and Ahmed et al. [23] proposed a new method based on the DDD for both the neonatal and pediatric populations. Despite the common attributable DDD drawbacks (weight variability, differences between established DDD and most used daily dose), all agree that the popularity of the DDD mainly originates from its general applicability and its advantage that comparison of the amount of drug use between different settings or drugs based on grouped dispensing data is possible without requiring utilization data at the individual patient level. In fact, some centers do not have the resources to measure other metrics; while DDD is plausible in most settings, being a real need to have additional options for children.

Focusing on the results of this study, in more than half of the selected antimicrobials, the final DDD could be established due to the high-power value. In those where the analysis could not be performed, but there was a high degree of concordance in the expert panel, we can consider selecting Phase I DDD and conducting studies with a higher power value.

It should be noted that the DDD values between both phases had a heterogeneous distribution. Thus, 65% of the DDD were higher in Phase II, 28% were superior in Phase I, and in the remaining 7%, the values were identical. This makes sense since if we analyze the demographic data of Phase I [12], we can observe that they are lower than those of Phase 2 (Table 2): 4.43 vs. 6.64 years and 17.08 vs. 25.70 kg. Therefore, the doses used in the validation phase will be generally higher, and this would explain why antibiotic consumption, expressed in DDD, is also higher. However, although these demographic differences have an impact on the value of the DDD, the truth is that the ages of the children included in the different phases were relatively similar, taking into account the wide range spanning the pediatric age. Therefore, this fact is indeed of great relevance since the ages obtained in both phases are consistent, making it possible to extrapolate the results obtained to the general pediatric population.

Phase I DDD was selected from 14 intravenous antimicrobials (48.3%) and 8 oral antimicrobials (57.1%) according to established criteria. In 12 intravenous (41.4%) and five oral (35.7%) antimicrobials, Phase II DDD was selected. The DDD calculated in the first stage was valid in more than 50% of the cases, and it was possible to obtain the pediatric DDD for 30 different antimicrobials (which translates into 39 DDD values depending on the formulation used), responding to the objectives of this study. Therefore, we can affirm that the methodology used is correct, being able to establish certain improvements in the design to try to obtain the DDD of those antimicrobials in which this has not been possible.

In the case of penicillin G and oral fosfomycin, the DDD could not be calculated due to an error in the collection of prescription data in Phase II. These errors may be due to the way these antimicrobials are expressed. Penicillin G can be dosed in IU or milligrams, and fosfomycin according to the sodium or trometamol presentation, which would justify the mistakes in data collection. In the rest of the antimicrobials (iv amoxicillin, iv cefuroxime, imipenem/cilastatin and cefadroxil), the optimal DDD could not be obtained according to the established selection criteria. Regarding amoxicillin and imipenem/cilastatin, although their DDD could not be determined because it had a power <80% and a degree of agreement among the panelists <75%, the truth is that DDD of both phases was similar, obtaining clinical differences <10%. In the case of cefuroxime, there were clinical differences, although the figure was not very high (12%). Finally, in relation to cefadroxil, the optimal DDD could not be defined due to the lack of consensus and the low number of prescriptions (n = 4). It should be noted that one of the patients received a very low dose due to his clinical situation, which greatly affected the value obtained. In this way, it is very probable that the DDD for these antimicrobials can be obtained in future studies, either by increasing the sample size or by creating a new panel of experts to try to agree on the doses of those most controversial drugs.

The DDD values obtained have not been compared with previous studies, given the lack of them using a similar methodology. However, if we calculate the medians of the different PDD (g) from the different age ranges obtained by D’Amore C et al. for the most frequently prescribed antibiotics [18], we observed that our DDD (g) are somewhat higher: piperacillin-tazobactam 5.1 vs. 3; meropenem 1 vs. 0.8, amoxicillin-clavulanate 1.7 vs. 0.7, amikacin 0.3 vs. 0.2, ceftriaxone 1.4 vs. 1, cefazolin 2 vs. 0.7, and gentamicin 0.14 vs. 0.05. Differences are explained by the calculation methods used. D’Amore C et al. calculated it by age intervals, while we have set a single DDD value for the entire pediatric population, something that we consider essential for the indicator to be applicable in clinical practice.

Regarding the weight used to calculate DDD, an adult is considered to be a person of 70 kg. In the case of the pediatric population, this value is probably more difficult to establish due to the great heterogeneity. In fact, we have not been able to identify in the literature a standard mean weight that can be established for this population. Liem TB et al. proposed a methodology to define DDD in the neonatal population and concluded that this methodology was not applicable in the pediatric population due to the large variation in body weight within this population [21]. For this reason, the method that we have designed to calculate the DDD in the pediatric population has also been based on real prescriptions that made it possible to obtain the grams of antibiotics consumed in the pediatric reference units of different hospitals.

The main limitations of our study include the lack of homogeneity in both the number of prescriptions collected for each antimicrobial and the number of samples provided by the participating hospitals. The lack of prescriptions for some antimicrobials does not reflect data loss; it could be due to the variability of antimicrobial use in the included centers. It would be interesting to propose more international multicenter studies to collect a truly representative sample of antimicrobials doses prescribed in routine clinical practice. However, despite the limitations related to sample size, all the antimicrobials included in the study were represented in the samples provided by the hospitals. It should be highlighted that the study has 40,575 children included in Phase I, and almost 5000 antimicrobial prescriptions were collected from real clinical practice for the validation process from hospitals with pediatric reference units. In addition, the participation of a group of experts to establish a consensus on the doses used to calculate the DDD with a high level of participation; and its subsequent clinical analysis confirms the validity of the results obtained.

Lastly, it must be considered that the use of pediatric DDD does not allow evaluation of the indication, but rather they, are a measure to estimate the consumption of antimicrobials considering the dose for the most common indication. Therefore, it does not accurately reflect doses in situations where antibiotic monitoring is required and doses are adjusted based on drug blood levels, or in clinical situations where patients require higher doses than usual: severe infection, central nervous system infection, sepsis, or otitis. All these situations can also occur in the adult population. Despite this, the DDD measure remains a gold standard numerator for comparing data on drug use and is used internationally [24].

As a result of this work, it has been possible to obtain pediatric DDD, emphasizing the importance of adding a specific indicator for this population that allows its global implementation. In addition, having a specific indicator will allow not only to evaluate the consumption of antimicrobials but also to analyze the effectiveness of the interventions carried out by ASP. A specific pediatric DDD could be used as an additional tool for ASP evaluation and monitoring in conjunction with other recommended metrics, and its practicality will allow its implementation when other metrics are not viable.

## Figures and Tables

**Table 1:** DDD selection criteria.

Power	DDD Selection	
>80%	Phase I	Phase II
	There are no significant differences (*p* > 0.01) + Clinical difference magnitude (<10%)	Statistically significant differences (*p* < 0.01) + Clinical difference magnitude (>10%)
	Statistically significant differences (*p* < 0.01) + Clinical difference magnitude (<10%)	
	There are no significant differences (*p* > 0.01) + Clinical difference magnitude (>10%) + Degree of agreement (≥75%)	
≤80%	Degree of agreement (≥75%)	NA

**Table 2:** Demographic characteristics of the pediatric patients and the treatments administered.

Demographic Characteristics	
Age, years	6.64 (4.6)
Weight, kg	25.70 (16.78)
Gender	
Female	1967 (41.1)
Male	2821 (58.9)
Antibiotic Administered	
Amikacin	64 (1.3)
Amoxicillin	152 (3.2)
Amoxicillin-clavulanic	999 (20.9)
Ampicillin	143 (3.0)
Amphotericin B liposomal	49 (1.0)
Azithromycin	90 (1.9)
Cefadroxil	4 (0.1)
Cefazolin	401 (8.4)
Cefepime	187 (3.9)
Cefixime	28 (0.6)
Cefotaxime	255 (5.3)
Ceftazidime	66 (1.4)
Ceftriaxone	161 (3.4)
Cefuroxime	107 (2.2)
Ciprofloxacin	96 (2.0)
Clarithromycin	23 (0.5)
Clindamycin	54 (1.1)
Cloxacillin	70 (1.5)
Daptomycin	4 (0.1)
Erythromycin	50 (1.0)
Fluconazole	121 (2.5)
Fosfomycin	9 (0.2)
Gentamicin	454 (9.5)
Imipenem-cilastatin	86 (1.8)
Levofloxacin	50 (1.0)
Linezolid	18 (0.4)
Meropenem	132 (2.8)
Metronidazole	420 (8.8)
Micafungin	45 (0.9)
Penicillin G	8 (0.1)
Piperacillin-tazobactam	133 (2.8)
Teicoplanin	119 (2.5)
Tobramycin	22 (0.5)
Vancomycin	168 (3.5)
**Route of administration**	
Oral	664 (13.5)
Intravenous	4144 (86.5)
Number of patients = 4788	

Qualitative variables are expressed as number (%); quantitative variables as mean (SD).

**Table 3:** Pediatric-defined daily doses of intravenously administered antimicrobials according to the results of data analysis.

Antimicrobials		Phase I DDD		Phase II DDD		Difference with Phase I DDD				DDD Differences between Phases		Power Value (>80%)	Difference Value (<10%)	Wilcoxon Test (>0.01)	Degree of Agreement (≥75%)	Selected DDD	Final DDD (g/day)
	N	Value (g/day)	Degree of Agreement	Median (g/day)	CI95%	Median	CI95%	Power (1-B) %	Wilcoxon Test	Median	CI95%						
GENTAMICIN	454	0.09	60	0.14	0.13, 0.15	−0.05	−0.06, 0.04	100	<0.001	56	44, 67	Yes	No	No	No	Phase II	0.14
CEFAZOLIN	401	1.71	80	2	2.00, 2.25	−0.29	−0.54, −0.29	100	<0.001	17	17, 32	Yes	No	No	Yes	Phase II	2
CEFTRIAXONE	161	0.85	50	1.4	1.15, 1.60	−0.55	−0.75, −0.30	100	<0.001	65	35, 88	Yes	No	No	No	Phase II	1.4
AMOXICILLIN-CLAVULANIC	866	1.71	90	1.8	1.74, 2.00	−0.09	−0.29, −0.03	100	<0.001	5	2, 17	Yes	Yes	No	Yes	Phase I	1.71
METRONIDAZOLE	395	0.51	100	0.9	0.84, 0.93	−0.39	−0.42, −0.33	100	<0.001	77	65, 82	Yes	No	No	Yes	Phase II	0.9
CEFEPIME	187	2.56	77.8	3	2.61, 3.00	−0.44	−0.44, −0.05	99.9	<0.001	17	2, 17	Yes	No	No	Yes	Phase II	3
CLOXACILLIN	65	1.71	90	3.5	2.40, 4.00	−1.79	−2.29, −0.69	99.9	<0.001	105	40, 134	Yes	No	No	Yes	Phase II	3.5
CLINDAMYCIN	54	0.51	60	0.74	0.51, 1.00	−0.225	−0.49, 0.00	99.9	<0.001	44	0, 96	Yes	No	No	No	Phase II	0.74
VANCOMYCIN	168	0.68	90	0.76	0.60, 0.96	−0.08	−0.28, 0.08	99.8	<0.001	12	−12, 41	Yes	No	No	Yes	Phase II	0.76
CIPROFLOXACIN	31	0.34	60	0.6	0.40, 0.80	−0.26	−0.46, 0.06	99.4	<0.001	77	18, 135	Yes	No	No	No	Phase II	0.6
AMPHOTERICIN B LIPO	49	0.05	66.7	0.08	0.05, 0.10	−0.025	−0.04, 0.00	99.3	<0.001	50	0, 90	Yes	No	No	No	Phase II	0.08
MEROPENEM	132	1.02	90	1.05	0.90, 1.26	−0.03	−0.24, 0.12	98.7	0.01	3	−12, 24	Yes	Yes	Yes	Yes	Phase I	1.02
TOBRAMYCIN	22	0.09	75	0.15	0.07, 0.36	−0.06	−0.27, 0.01	96	0.009	67	−17, 300	Yes	No	No	Yes	Phase II	0.15
ERYTHROMYCIN	26	0.68	100	1.1	0.52, 1.26	−0.42	−0.58, 0.16	88.7	0.006	62	−24, 85	Yes	No	No	Yes	Phase II	1.1
AMPICILLIN	143	1.71	90	1.6	1.34, 2.00	0.11	−0.29, 0.37	84.6	0.217	−6	−22, 17	Yes	Yes	Yes	Yes	Phase I	1.71
FLUCONAZOLE	38	0.1	100	0.1	0.06, 0.18	0	−0.07, 0.04	81.6	0.058	0	−40, 80	Yes	Yes	Yes	Yes	Phase I	0.1
AMIKACIN	64	0.26	88.9	0.25	0.19, 0.32	0.01	−0.06, 0.06	71.5	0.556	−3,8	−25, 23	No	Yes	NA	Yes	Phase I	0.26
AMOXICILLIN	45	1.37	57.1	1.44	1.35, 1.50	−0.07	−0.13, 0.02	70.9	0.227	5	−1, 9	No	Yes	NA	No	-	-
MICAFUNGIN	45	0.03	87.5	0.03	0.02, 0.04	−0.001	−0.01, 0.01	64.5	0.057	3	−20, 50	No	Yes	NA	Yes	Phase I	0.03
AZITHROMYCIN	15	0.17	100	0.18	0.51, 1.00	−0.005	−0.33, 0.04	60.4	0.200	3	−26, 194	No	Yes	NA	Yes	Phase I	0.17
TEICOPLANIN	119	0.17	77.8	0.15	0.10, 0.17	0.02	0.00, 0.07	45.5	0.656	−12	−41, 3	No	No	NA	Yes	Phase I	0.17
CEFUROXIME	66	1.71	60	1.5	0.90, 2.25	0.21	−0.54, −0.81	34.3	0.668	−12	−47, 32	No	No	NA	No	-	-
LINEZOLID	11	0.51	100	0.45	0.21, 1.20	0.06	−0.69, 0.30	32.5	0.308	−12	−59, 135	No	No	NA	Yes	Phase I	0.51
IMIPENEM/ CILASTATIN	86	1.71	66.7	1.6	1.20, 1.92	0.11	−0.21, 0.51	31.2	0.917	−6	−30, 12	No	Yes	NA	No	-	-
PIPERACILLIN-TAZOBACTAM	133	5.12	100	3.9	3.00, 5.00	1.22	0.12, 2.12	29.3	0.678	−24	−41, −2	No	No	NA	Yes	Phase I	5.12
CEFTAZIDIME	66	2.56	80	2.55	1.86, 3.00	0.01	−0.44, 0.70	28	0.548	0	−27, 17	No	Yes	NA	Yes	Phase I	2.56
LEVOFLOXACIN	35	0.34	75	0.4	0.30, 0.50	−0.06	−0.16, 0.04	20.8	0.744	18	−12, 47	No	No	NA	Yes	Phase I	0.34
DAPTOMYCIN	4	0.14	85.7	0.17	NA	−0.025	NA	14.1	0.625	18	NA	No	No	NA	Yes	Phase I	0.14
CEFOTAXIME	255	2.56	80	2	1.60, 2.40	0.56	0.16, 0.96	6.7	0.048	−22	−38, −6	No	No	NA	Yes	Phase I	2.56

CI: Confidence Interval.

**Table 4:** Pediatric-defined daily doses of orally administered antimicrobials according to the results of data analysis.

Antimicrobials		Phase I DDD		Phase II DDD		Difference with Phase I DDD				DDD Differences between Phases		Power Value (>80%)	Difference Value (<10%)	Wilcoxon Test (>0.01)	Degree of Agreement (≥75%)	Selected DDD	Final DDD (g/day)
	N	Value (g/day)	Degree of Agreement	Median (g/day)	CI95%	Median	CI95%	Power (1-B) %	Wilcoxon Test	Median	CI95%						
AMOXICILLIN-CLAVULANIC	133	0.68	40	0.9	0.81, 1.05	−0.22	−0.37, −0.13	100	<0.001	32.4	19, 54	Yes	No	No	No	Phase II	0.9
CIPROFLOXACIN	65	0.34	100	0.5	0.50, 0.80	−0.16	−0.46, −0.16	99.9	<0.001	47.1	47, 135	Yes	No	No	Yes	Phase II	0.5
CEFUROXIME	41	0.26	10	0.5	0.28, 0.58	−0.24	−0.32, 0.02	99.8	<0.001	92.3	−8, 123	Yes	No	No	No	Phase II	0.5
LEVOFLOXACIN	15	0.17	42.9	0.26	0.22, 0.50	−0.09	−0.33, −0.05	99.3	0.001	529	29, 194	Yes	No	No	No	Phase II	0.26
FLUCONAZOLE	83	0.1	100	0.1	0.10, 0.10	0	0.00, 0.00	98.2	0.022	0	0, 0	Yes	Yes	Yes	Yes	Phase I	0.1
AMOXICILLIN	107	0.85	50	0.9	0.83, 1.05	−0.05	−0.20, 0.02	95.1	0.007	5.9	−3, 24	Yes	Yes	No	No	Phase I	0.85
CEFIXIME	28	0.14	100	0.19	0.12, 0.40	−0.045	−0.26, 0.02	88.8	0.021	32.1	−14, 186	Yes	No	Yes	Yes	Phase I	0.14
CLARITHROMYCIN	23	0.26	100	0.36	0.30, 0.40	−0.1	−0.14, 0.04	83.1	0.004	38.5	15, 54	Yes	No	No	Yes	Phase II	0.36
AZITHROMYCIN	75	0.17	100	0.14	0.12, 0.20	0.03	−0.03, 0.04	59.1	0.594	−17.6	−26, 18	No	No	NA	Yes	Phase I	0.17
METRONIDAZOLE	25	0.51	100	0.65	0.38, 1.00	−0.14	−0.49, 0.14	53.7	0.148	27.5	−26, 96	No	No	NA	Yes	Phase I	0.51
LINEZOLID	7	0.51	100	0.66	0.24, 1.20	−0.15	−0.69, 0.27	41.7	0.148	29.4	−53, 135	No	No	NA	Yes	Phase I	0.51
CLOXACILLIN	5	1.71	80	3	NA	−1.29	NA	17.1	0.313	75.4	NA	No	No	NA	Yes	Phase I	1.71
ERYTHROMYCIN	24	0.68	100	0.28	0.10, 0.80	0.4	−0.12, 0.58	16.1	0.174	−58.8	−85, 18	No	No	NA	Yes	Phase I	0.68
CEFADROXIL	4	0.51	66.7	0.16	NA	0.355	NA	13.1	0.875	−69.6	NA	No	No	NA	No	-	-

CI: Confidence Interval.

**Table 5:** Selection of DDD for each antibiotic based on the established criteria.

Power	DDD Selection	
**>80%**	**Phase I**	**Phase II**
	No statistically significant differences + no clinical difference magnitude Intravenous: meropenem, ampicillin and fluconazole Oral: fluconazole	Statistically significant differences + clinical difference magnitude Intravenous: cefazolin, metronidazole, cefepime, cloxacillin, vancomycin, tobramycin and erythromycin, gentamicin, ceftriaxone, clindamycin, ciprofloxacin, amphotericin B. Oral: ciprofloxacin, clarithromycin, amoxicillin-clavulanic, cefuroxime, levofloxacin
	Statistically significant differences + no clinical difference magnitude Intravenous: amoxicillin-clavulanic Oral: amoxicillin	
	No statistically significant differences + clinical difference magnitude + degree of agreement Oral: cefixime	
**≤80%**	Degree of agreement (≥75%) Intravenous: amikacin, micafungin, azithromycin, teicoplanin, linezolid, piperacillin-tazobactam, ceftazidime, levofloxacin, daptomycin, cefotaxime Oral: azithromycin, metronidazole, linezolid, cloxacillin, erythromycin	

## Data Availability

Not applicable.

## Supplementary Materials

The following supporting information can be downloaded at: https://www.mdpi.com/article/10.3390/antibiotics12020276/s1, Table S1: Pediatric DDD.
