# Supplementary material for: Development of Antimicrobial Defined Daily Dose (DDD) for the Pediatric Population
Source: Antibiotics (Basel). 2023 Jan 31;12(2):276. doi: 10.3390/antibiotics12020276 (PMC9952639; doi:10.3390/antibiotics12020276)
Supplement: Supplementary file 1 [file antibiotics-12-00276-s001.zip › antibiotics-2183195-supplementary.pdf]

Supplementary File 1.

Table S1. Pediatric DDD

ANTIMICROBIALS	FINAL DDD (g/day)	
	Intravenous	Oral
AMIKACIN	**0.26**	-
AMOXICILLIN	**-**	**0.85**
AMOXICILLIN-CLAVULANIC	**1.71**	**0.9**
AMPHOTERICIN B LIPO	**0.08**	-
AMPICILLIN	**1.71**	-
AZITHROMYCIN	**0.17**	**0.17**
CEFAZOLIN	**2**	-
CEFEPIME	**3**	-
CEFIXIME	-	**0.14**
CEFOTAXIME	**2.56**	-
CEFTAZIDIME	**2.56**	-
CEFTRIAXONE	**1.4**	-
CEFUROXIME	**-**	**0.5**
CIPROFLOXACIN	**0.6**	**0.5**
CLARITHROMYCIN	-	**0.36**
CLINDAMYCIN	**0.74**	-
CLOXACILLIN	**3.5**	**1.71**
DAPTOMYCIN	**0.14**	-
ERYTHROMYCIN	**1.1**	**0.68**
FLUCONAZOLE	**0.1**	**0.1**
GENTAMICIN	**0.14**	-
IMIPENEM/CILASTATIN	**-**	-
LEVOFLOXACIN	**0.34**	**0.26**
LINEZOLID	**0.51**	**0.51**
MEROPENEM	**1.02**	-
METRONIDAZOLE	**0.9**	**0.51**
MICAFUNGIN	**0.03**	-
PIPERACILLIN-TAZOBACTAM	**5.12**	-
TEICOPLANIN	**0.17**	-
TOBRAMYCIN	**0.15**	-
VANCOMYCIN	**0.76**	-
